# Frontale Hypopigmentierung mit bandförmiger Alopezie

**DOI:** 10.1007/s00105-022-04976-9

**Published:** 2022-03-28

**Authors:** Daisy Kopera, Lorenzo Cerroni, Ralph Trüeb

**Affiliations:** 1 Klinik für Dermatologie, Medizinische Universität Graz, Auenbruggerplatz 8, 8036 Graz, Österreich; 2 Haarcenter Wallisellen, Wallisellen, Schweiz

## Anamnese

Eine 56-jährige Patientin in gutem Allgemeinzustand präsentiert sich mit einer bandförmigen Hypopigmentierung parallel zur frontalen Haarlinie, die sich über 18 Monate langsam fortschreitend entwickelte. Bis auf 1999 Hysterektomie mit Adnexektomie und 2018 Labiensynechie bei Verdacht auf Lichen sclerosus atrophicans ohne histologischen Nachweis.

## Klinischer Befund

**Figure 1:**
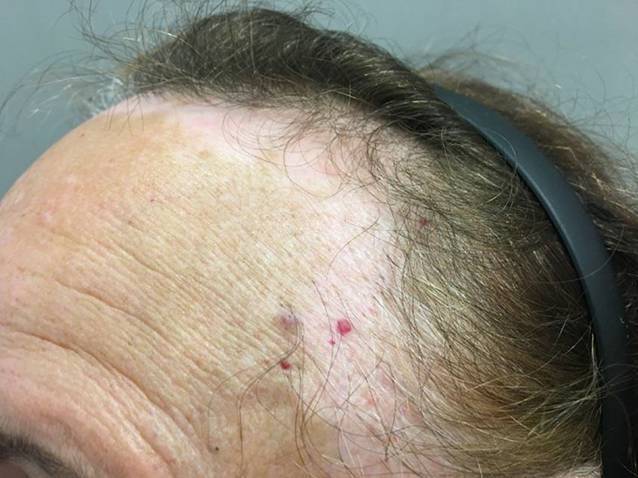

**Figure 2:**
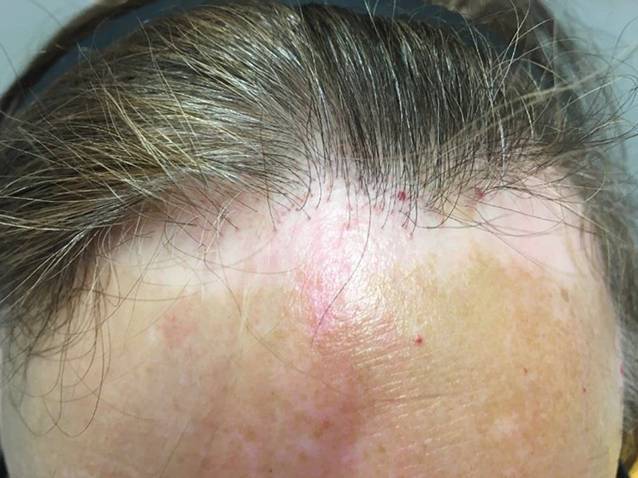

Es zeigt sich eine etwa 2 cm breite, dem frontalen Haaransatz parallel verlaufende Depigmentierung. Die Haut wirkt etwas atroph und zeigt einzelne eruptive Angiome (Abb. 1). Die vordere Haaransatzlinie hat sich ca. 2 cm zurückgebildet, es zeigen sich einzeln stehende Haare, fehlende Vellushaare und ein perifollikuläres Erythem mit diskreten Schuppenkrausen (Abb. 2). Die übrige Kopfhaut und Haardichte sind regelrecht. Die Augenbrauen sind unauffällig. Das restliche Integument zeigt keine nennenswerten Hautveränderungen.

## Labor

Routinelabor inklusive Immunserologie und Blutbild mit Differenzialblutbild sind altersentsprechend im Normbereich, die indirekte Immunfluoreszenz ist negativ. Auf die direkte Immunfluoreszenz wurde aufgrund unsicherer Aussage bewusst verzichtet, da falsch positive Reaktionen in lichtexponierten Arealen, v. a. im Gesicht, relativ oft vorkommen [1].

## Histologie

Atrophe Epidermis mit Zeichen einer Interface-Dermatitis. Mäßig dichte lymphoidzellige Entzündungsinfiltrate. Immunhistologisch in der CD123-Färbung zeigen sich vereinzelt positive plasmazytoide dendritische Zellen.

## Wie lautet Ihre Diagnose?

**Diagnose:** Kutaner Lupus erythematodes unter dem Bild einer frontalen fibrosierenden Alopezie.

## Therapie

Nach Vorliegen eines unauffälligen ophthalmologischen Befundes und unauffälliger Glucose-6-Phosphat-Dehydrogenase im Serum wurde eine systemische Behandlung mit Hydroxychloroquinsulfat 5 mg/kg Körpergewicht 1‑mal täglich eingeleitet.

## Klinische Differenzialdiagnosen

- Vitiligo
- Extragenitaler Lichen sclerosus
- Postmenopausale frontale fibrosierende Alopezie
- Kutaner Lupus erythematodes unter dem Bild einer frontalen fibrosierenden Alopezie

Die Differenzialdiagnosen Vitiligo und extragenitaler Lichen sclerosus können aufgrund des histologischen Befundes ausgeschlossen werden.

## Definition und Hintergrund

Ob der Australier Steven Kossard 1994 [2] oder der schwedische Arzt Axel Munthe [3] das Krankheitsbild der postmenopausalen frontalen fibrosierenden Alopezie (PFFA) erstmals beschrieben haben, sei dahingestellt. Faktum ist, dass die exakte Diagnose einer vernarbenden Alopezie Probleme bereiten kann [4]. Im vorliegenden Fall lenkt die Hypopigmentierung auf den ersten Blick vom Wesentlichen ab, denn bei genauerer Inspektion wird eine assoziierte follikuläre Entzündung mit selektivem Schwund von Haarfollikeln ersichtlich. Die bandförmige Verteilung der follikulären entzündlich atrophisierenden Alopezie lässt in erster Linie an eine frontale fibrosierende Alopezie denken. Zudem schließt die histologische Untersuchung den extragenitalen Lichen sclerosus aus. Dafür zeigten sich eine Histologie und indirekte Immunfluoreszenzuntersuchung, die vereinbar waren mit einem kutanen Lupus erythematodes. Postläsionelle Hypopigmentierung im Rahmen von kutanem Lupus erythematodes ist bekannt. Während die PFFA in der Originalpublikation von Kossard histologisch einen Lichen planopilaris zeigte, sind inzwischen Fälle von kutanem Lupus erythematodes unter dem klinischen Erscheinungsbild einer PFFA publiziert worden [5]. Es stellt sich damit die Frage nach der eigenständigen nosologischen Entität der PFFA, oder ob es sich bei der PFFA nicht um eine besondere Präsentationsform entzündlich narbiger Alopezien wie Lichen planopilaris und Lupus erythematodes handelt [6, 7]. Nach ausführlichem Studium der entsprechenden Literatur haben wir uns im Einklang mit den Befunden für die Diagnose eines kutanen Lupus erythematodes unter dem Bild einer PFFA entscheiden. Als Therapie der Wahl des kutanen Lupus erythematodes haben wir uns für Hydroxychloroquinsulfat entschieden.

## Fazit für die Praxis

Vernarbende Alopezien können auch für erfahrene Haar- und Kopfhautspezialisten eine Herausforderung darstellen. Eine Biopsie aus dem betroffenen Areal ist für eine exakte Diagnosestellung unumgänglich. Dies trifft auch auf die sonst typische Präsentationsform der PFFA zu.
